# Association of Traumatic Injury With Adverse Pregnancy Outcomes in Taiwan, 2004 to 2014

**DOI:** 10.1001/jamanetworkopen.2021.7072

**Published:** 2021-04-20

**Authors:** Chih-Wei Pai, Bayu Satria Wiratama, Hsiao-Yu Lin, Ping-Ling Chen

**Affiliations:** 1 Graduate Institute of Injury Prevention and Control, College of Public Health, Taipei Medical University, Taipei, Taiwan; 2 Department of Epidemiology, Biostatistics and Population Health, Faculty of Medicine, Public Health and Nursing, Universitas Gadjah Mada, Yogyakarta City, Indonesia; 3 Department of Urology, Taipei Medical University Hospital, Taipei, Taiwan

## Abstract

**Importance:**

Literature has suggested that trauma among pregnant women is associated with an increased risk of adverse pregnancy outcomes. However, limited research has investigated the association of trauma with adverse pregnancy outcomes by using a national data set.

**Objective:**

To investigate the association between traumatic injury and adverse pregnancy outcomes.

**Design, Setting, and Participants:**

This population-based cohort study of pregnant women in Taiwan linked 3 data sets, the Taiwan Birth Registry, Household Registration Information, and National Health Insurance Research Database, from January 1, 2004, through December 31, 2014. Data, including the characteristics of pregnant women and infants, were extracted from the Taiwan Birth Registry data set; to obtain trauma data, this data set and the Household Registration Information data set were collectively linked to National Health Insurance Research Database data. The combined data set was analyzed from January to July 2019. Adverse pregnancy outcomes and premature delivery were defined using *International Classification of Diseases, Ninth Revision, Clinical Modification* codes.

**Exposures:**

The primary exposures of this study were 2 clinical variables related to injury during pregnancy: medical treatment in the emergency department (yes or no) and hospitalization (yes or no).

**Main Outcomes and Measures:**

The main outcome variable was adverse pregnancy outcomes, and the secondary outcome variable was premature delivery. Multivariate logistic regression models were used to investigate the association of injuries with adverse pregnancy outcomes after controlling for demographic characteristics and other pregnancy-related variables.

**Results:**

A total of 2 973 831 pregnant women (2 475 805 [83.3%] aged 20-34 years) were enrolled between 2004 and 2014, of whom 59 681 (2.0%) sought medical treatments due to injuries. Results of multivariate logistic regression models showed that women receiving emergency treatments more than once were 1.08 times as likely (adjusted odds ratio, 1.08; 95% CI, 1.05-1.10) to have adverse pregnancy outcomes than women who received no emergency treatment. Women with injury-related hospitalization were 1.53 times more likely (adjusted odds ratio, 1.53; 95% CI, 1.41-1.65) to have adverse pregnancy outcomes than women who did not sustain injuries. Furthermore, recurrent injuries were associated with a 572% increase in odds of premature delivery (adjusted odds ratio, 6.72; 95% CI, 2.86-15.80).

**Conclusions and Relevance:**

In this study, trauma among pregnant women was associated with an increased risk of adverse pregnancy outcomes, as were hospitalization and emergency department visits due to injury.

## Key Points

**Question:**

Do differences exist in the risk of adverse pregnancy outcomes among women who had emergency treatment or hospitalization due to injuries compared with those who did not?

**Findings:**

In this cohort study of 2 973 831 pregnant women, emergency treatment for injuries was associated with an increased risk of adverse pregnancy outcomes. Women with injury-related hospitalization were also at an increased risk of adverse pregnancy outcomes.

**Meaning:**

This study found that pregnant women who sustained injuries requiring hospitalization or emergency department visits exhibited higher risks of adverse pregnancy outcomes, including premature delivery.

## Teaser

This population-based cohort study examines the association between traumatic injury and adverse pregnancy outcomes in Taiwan.

## Introduction

Trauma is estimated to complicate 6% to 7% of all pregnancies and to be one of the leading nonobstetric causes of maternal death during pregnancy.^1,2,3^ It is also associated with an increased risk of fetal death, with 3 to 7 fetal deaths per 100 000 live births.^4^ Moreover, severe trauma is associated with a higher risk of fetal death.^5^

Studies have suggested a direct association between trauma and maternal and fetal death during pregnancy. Motor vehicle collisions during pregnancy were indicated to be a significant risk factor for maternal and fetal deaths.^6^ Pregnant women involved in burn incidents had a higher risk of death, with sepsis and smoke inhalation as the major risk factors.^7,8^ Penetration trauma during pregnancy was also associated with increased fetal mortality and hospital stay.^2^

Apart from fetal deaths, trauma has also been associated with other adverse pregnancy outcomes. Compared with control participants who did not experience trauma, women with trauma had increased odds of placental abruption, uterine rupture, and fetal distress.^9^ Similarly, Schiff and Holt^10^ indicated that women who were severely and nonseverely injured in motor vehicle collisions had higher risks of placental abruption and cesarean delivery. Weiss et al^11^ reported that women with injury-related emergency department visits were more likely to experience placental abruption and cesarean delivery. In Taiwan, women with severe and minor injuries also had higher odds of preterm labor.^5^

Relevant literature has suggested that trauma among pregnant women is associated with an increased risk of adverse outcomes in pregnancy and preterm labor.^5,9,10,11^ However, previous studies mostly have used only small data sets (nonnational data sets) and adjusted the final model with limited clinical variables. While exceptional studies, such as that by Cheng et al,^5^ have used 1 national data set (ie, the National Health Insurance Research Database data set), our study investigated the association of trauma-related factors with adverse pregnancy outcomes by using 3 national data sets in Taiwan. Furthermore, we also included broad research variables. First, we estimated 3 categoric levels of hospitalization and emergency treatment: none, once, and more than once. Next, we adjusted the final model by using different types of comorbidities, such as anemia, heart disease, lung disease, diabetes, and kidney disease. Finally, we adjusted the model with different types of pregnancy treatments, such as amniocentesis, labor induction, and cervical cerclage surgery.

## Methods

### Data Sources

The current study used 3 national data sets—namely, the Taiwan Birth Registry, Household Registration Information, and the National Health Insurance Research Database—from January 1, 2004, through December 31, 2014. The Taiwan Birth Registry data set contains data on demographic characteristics (eg, age at delivery, education level, marital status), birth characteristics (eg, congenital anomalies, gestational age, birth weight, stillbirth), and Apgar scores.^12,13^ The Household Registration Information data set features the household and personal characteristics of people living in Taiwan, such as age, sex, race, income, and residential area. The National Health Insurance Research Database comprises medical claims, covering the medical records of nearly all people (>99%) living in Taiwan. The National Health Insurance Research Database contains data on patients’ age, sex, and disease diagnosis (in accordance with the *International Classification of Diseases, Ninth Revision, Clinical Modification* [*ICD-9-CM*]). The 3 data sets were linked with patients’ encrypted identification numbers to create 1 master database. This study was approved by the institutional review board of Taipei Medical University. No individual patient or casualty can be identified, and therefore informed consent was waived. This study followed the Strengthening the Reporting of Observational Studies in Epidemiology (STROBE) reporting guideline.^14^

### Definition of Variables

The main outcome variable was adverse pregnancy outcomes, and the most common adverse pregnancy outcome in Taiwan, premature delivery, was selected as the secondary outcome. We defined adverse pregnancy outcomes as 1 of the following: birth defects (*ICD-9-CM* codes 740-759), birth weight less than 2500 g (*ICD-9-CM* codes 765.01-765.08), premature delivery (*ICD-9-CM* codes 765.21-765.28), or stillbirth (*ICD-9-CM* code 779.9); a normal pregnancy outcome was defined as a full-term live birth with no birth defects and birth weight greater than 1500 g. Premature delivery was defined as delivery before the start of the 37th week of pregnancy; full-term delivery was defined as delivery at the 37th week of pregnancy or later.

For this study, basic demographic data were collected, including maternal age (<20, 20-34, or >34 years), race (Aboriginal and Non-Indigenous, given that Aboriginal populations were hypothesized to have a higher injury prevalence), and nationality (Taiwanese or other nationality). Women younger than 20 years or older than 34 years have higher risks of complications during pregnancy.^14^ We also collected data on pregnant women’s income status (low, middle, and high income as well as nonself-income) and residential area (highly urbanized area, moderately urbanized area, new city, general city, and rural, agricultural, or aging city). Residential area was defined according to research by Cavazos-Rehg et al.^15^

We also collected the following 3 clinical variables: other disease or substance use during pregnancy (yes or no), labor complications (yes or no), and specific medical treatment during pregnancy (yes or no). We defined pregnant women as having other diseases or engagement in substance use during pregnancy if they had at least 1 *ICD-9* code for anemia, heart disease, lung disease, diabetes, infections (eg, syphilis), gestational diabetes, excessive or insufficient amniotic fluid, high blood pressure, pregnancy toxemia, incomplete cervix, history of giving birth to newborns weighing at least 4000 g, history of premature or underweight neonates, kidney disease, Rh factor allergy, autoimmune disease (eg, lupus erythematosus), prenatal bleeding, smoking during pregnancy, alcohol use disorder, or drug addiction during pregnancy. Drug addiction was defined as pregnant women who had at least 1 *ICD-9* code related to substance use disorders (292.0-292.9, 304.00-304.93, 305.00-305.93, 648.30-648.34, 968.5, 969.9, 965.00-965.09, E854.1, E939.6, E850.0, E935.0, V654.2, or E938.5). We defined labor complications as any of the following medical conditions experienced during pregnancy: fever, fecal material in amniotic fluid, early water breakage, early placental detachment, placenta previa, massive bleeding, spasms during childbirth, acute labor, prolonged labor, abnormal labor progression, breech or fetal position, cephalopelvic disproportion, complications related to umbilical cord prolapse, anesthesia, or fetal distress. Specific medical treatment requirements during pregnancy were defined as 1 of the following: amniocentesis, villi examination, labor induction, birth induction, fetal placement, cervical cerclage surgery, or laparotomy.

Two clinical variables related to injury during pregnancy were used in the study: emergency medical treatment (yes or no) and hospitalization (yes or no). Data related to injury status were extracted from the National Health Insurance Research Database by using *ICD-9-CM* N codes 800 to 999. Women were defined as having received emergency medical treatment because of injury if they received an injury code diagnosis during treatment in the emergency department and as having required hospitalization owing to injury if they had records of hospitalization and injury diagnosis with *ICD-9-CM* codes.

### Statistical Analysis

First, we described the distribution of adverse pregnancy outcomes and premature delivery with demographic, clinical, and injury-related variables by using absolute numbers and percentages. Next, we applied simple logistic regression to analyze the association between study outcomes (adverse pregnancy outcomes and premature delivery) and risk factors. We used *P* < .20 as a cutoff point to determine risk factors to be included in multivariable analysis, consistent with previous research.^16,17,18,19,20^ We also chose the covariates in accordance with studies that confirmed the association between the covariates and outcome variables. For the multivariate analysis, we used multiple logistic regression with a backward selection to calculate the adjusted odds ratio (AOR) for each risk factor. We conducted 2 separate bivariate and multivariate analyses for adverse pregnancy outcomes and premature delivery. We assessed multicollinearity by using Cramer V and the χ^2^ independence test. This study used 95% CIs and an α value of .05. A 2-sided hypothesis test was used for all statistical analysis in this study, which was performed with Stata version 15 (StataCorp).

## Results

### Characteristics of Pregnant Women

Table 1 illustrates the characteristics of women in this study. Of 2 973 831 women, 392 619 (13.2%) had adverse pregnancy outcomes. Most women (2 475 805 [83.3%]) were aged 20 to 34 years. Most women (2 518 567 [84.7%]) did not require any specific medical treatment during pregnancy and delivery. Overall, 1 365 084 women (45.9%) had other diseases during pregnancy, such as heart disease, diabetes, lung disease, or kidney disease. Most women were non-Indigenous (2 875 568 [96.7%]), were Taiwanese (2 511 427 [90.4%]), received no emergency medical treatment due to injury during pregnancy (2 911 379 [97.9%]), and required no hospitalization due to injuries during pregnancy (2 969 718 [99.9%]). A total of 625 125 women (21.1%) resided in highly urbanized areas, whereas 301 808 (10.1%) resided in rural, agricultural, or aging cities. Furthermore, 505 641 women (17.0%) were without self-income, and 1 211 937 (40.7%) were in the middle-income group.

**Table 1.** Distribution of Risk Factors for Adverse Pregnancy Outcomes^a^

Characteristic	No. (%)		
	No adverse pregnancy outcome (n = 2 581 212)	Adverse pregnancy outcome (n = 392 619)	Total (N = 2 973 831)
Maternal age, y			
<20	62 666 (82.5)	13 320 (17.5)	75 986 (2.6)
20-34	2 166 862 (87.5)	308 943 (12.5)	2 475 805 (83.3)
≥35	351 684 (83.3)	70 356 (16.7)	422 040 (14.1)
Residential area			
Highly urbanized area	545 157 (87.2)	79 968 (12.8)	625 125 (21.1)
Moderately urbanized area	794 075 (87.0)	118 851 (13.0)	912 926 (30.8)
New city	594 523 (86.8)	90 372 (13.2)	684 895 (23.1)
General city	382 719 (86.5)	59 478 (13.5)	442 197 (14.9)
Rural, agricultural, or aging city	258 759 (85.7)	43 049 (14.3)	301 808 (10.1)
Race			
Non-Indigenous	2 500 858 (87.0)	374 710 (13.0)	2 875 568 (96.7)
Aboriginal	80 354 (81.8)	17 909 (18.2)	98 263 (3.3)
Nationality			
Taiwanese	2 171 781 (86.5)	339 646 (13.5)	2 511 427 (90.4)
Non-Taiwanese	240 164 (89.9)	27 127 (10.1)	267 291 (9.6)
Income			
Low	556 165 (85.5)	94 043 (14.5)	650 208 (21.9)
Middle	1 053 691 (86.9)	158 246 (13.1)	1 211 937 (40.7)
High	529 305 (87.3)	76 740 (12.7)	606 045 (20.4)
No self-income	442 051 (87.4)	63 590 (12.6)	505 641 (17.0)
Other diseases or substance use during pregnancy^b^			
Yes	1 150 322 (84.3)	214 762 (15.7)	1 365 084 (45.9)
No	1 430 890 (88.9)	177 857 (11.1)	1 608 747 (54.1)
Labor complications^c^			
Yes	356 528 (78.7)	96 239 (21.3)	452 767 (15.2)
No	2 224 684 (88.2)	296 380 (11.8)	2 521 064 (84.8)
Specific medical treatment during pregnancy and delivery^d^			
Yes	368 675 (81.0)	86 589 (19.0)	455 264 (15.3)
No	2 212 537 (87.8)	306 030 (12.2)	2 518 567 (84.7)
Emergency medical treatment due to injury during pregnancy			
Yes	53 307 (85.4)	9145 (14.6)	62 452 (2.1)
Once	51 033 (85.5)	8648 (14.5)	59 681 (2.0)
>Once	2274 (82.1)	497 (17.9)	2771 (0.1)
No	2 527 905 (86.8)	383 474 (13.2)	2 911 379 (97.9)
Ever hospitalized due to injury during pregnancy			
Yes	3286 (79.9)	827 (20.1)	4113 (0.138)
Once	3219 (79.9)	809 (20.1)	4028 (0.135)
>Once	67 (78.8)	18 (21.2)	85 (0.003)
No	2 577 926 (86.8)	391 792 (13.2)	2 969 718 (99.9)

^a^ Adverse pregnancy outcomes include birth defects, low birth weight (<2500 g), premature delivery (at <37 weeks), or stillbirth; full-term live births with no birth defects and birth weight greater than 2500 g were considered to signify no adverse pregnancy outcomes.

^b^ Includes anemia, heart disease, lung disease, diabetes, infections (eg, syphilis), gestational diabetes, excessive or insufficient amniotic fluid, high blood pressure, pregnancy toxemia, incomplete cervix, a history of producing newborns weighing at least 4000 g, a history of producing premature newborns or underweight infants, kidney disease, Rh factor allergy, autoimmune disease (eg, lupus erythematosus), prenatal bleeding, smoking during pregnancy, alcoholism, and drug addiction during pregnancy.

^c^ Includes fever, fecal material in amniotic fluid, early water breakage, early placental detachment, placenta previa, massive bleeding, spasms during childbirth, acute labor, prolonged labor, abnormal labor progression, breech or fetal position, cephalopelvic disproportion, complications related to umbilical cord prolapse, anesthesia, and fetal distress.

^d^ Includes amniocentesis, villi examination, labor induction, birth induction, fetal placement, cervical cerclage surgery, and laparotomy.

### Distribution of Factors Associated With Adverse Pregnancy Outcomes

Table 1 also presents the distribution of risk factors for adverse pregnancy outcomes. Adverse pregnancy outcomes were more prevalent among women who were Taiwanese (339 646 [13.5%]) vs other nationalities (27 127 of 267 291 [10.1%]), were Aboriginal (17 909 of 98 263 [18.2%]) vs non-Indigenous (374 710 of 2 875 568 [13.0%]), were younger than 20 years (13 320 of 75 986 [17.5%]) vs aged 20 to 34 years (308 943 of 2 475 805 [83.3%]), and had low income (94 043 of 650 208 [14.5%]) vs high income (76 740 of 606 045 [12.7%]). The proportion of adverse outcomes was higher among women who had other diseases or engaged in substance use during pregnancy (214 762 of 1 365 084 [15.7%]) and those who had labor complications (96 239 of 452 767 [21.3%]) than among those who had no other diseases or engagement in substance use during pregnancy (177 857 of 1 608 747 [11.1%]) or those who had no labor complications (296 380 of 2 521 064 [11.8%]). Women who received emergency medical treatment due to injuries (9145 of 62 452 [14.6%]) had higher risks of adverse outcomes than those who required none (383 474 of 2 911 379 [13.2%]). Women requiring specific medical treatments during pregnancy and delivery (86 589 of 455 264 [19.0%]) had higher risks of adverse pregnancy outcomes than did those requiring none (306 030 of 2 518 567 [12.2%]).

### Distribution of Factors Associated With Premature Delivery

Table 2 shows the distribution of risk factors for premature delivery, such as age, area, income, and other diseases or substance use during pregnancy. This study showed that a higher proportion of women who had labor complications (75 134 of 285 419 [17.4%]) delivered prematurely than those who had none (210 285 [8.6%]). A higher proportion of women who had other diseases or engagement in substance use during pregnancy (161 082 [12.3%]) delivered prematurely than those who had none (124 337 [8.0%]). Premature delivery was more prevalent among women who received emergency medical treatment owing to injury during pregnancy (6359 [10.7%]) than among those who did not (279 060 [9.9%]). Among women hospitalized due to injuries, 610 (15.7%) delivered prematurely. A total of 70 190 women (16.0%) who received specific medical treatment during pregnancy and delivery had premature deliveries.

**Table 2.** Distribution of Risk Factors for Premature Delivery

Characteristic	No. (%)	
	Full-term delivery (n = 2 581 212)	Premature delivery (n = 285 419)
Maternal age, y		
<20	62 666 (87.2)	9180 (12.8)
20-34	2 166 862 (90.7)	221 376 (9.3)
≥35	351 684 (86.5)	54 863 (13.5)
Residential area		
Highly urbanized area	545 157 (90.4)	57 970 (9.6)
Moderately urbanized area	794 075 (90.2)	86 676 (9.8)
New city	594 523 (90.0)	66 323 (10.0)
General city	382 719 (89.9)	43 035 (10.1)
Rural, agricultural, or aging city	258 759 (89.4)	30 727 (10.6)
Race		
Non-Indigenous	2 500 858 (90.2)	272 813 (9.8)
Aboriginal	80 354 (86.4)	12 606 (13.6)
Nationality		
Taiwanese	2 171 781 (89.8)	247 315 (10.2)
Non-Taiwanese	240 164 (92.6)	19 195 (7.4)
Income		
Low	556 165 (89.1)	67 866 (10.9)
Middle	1 053 691 (90.1)	115 369 (9.9)
High	529 305 (90.3)	56 782 (9.7)
No self-income	442 051 (90.7)	45 402 (9.3)
Other diseases or substance use during pregnancy^a^		
Yes	1 150 322 (87.7)	161 082 (12.3)
No	1 430 890 (92.0)	124 337 (8.0)
Labor complications^b^		
Yes	356 528 (82.6)	75 134 (17.4)
No	2 224 684 (91.4)	210 285 (8.6)
Specific medical treatment during pregnancy and delivery^c^		
Yes	368 675 (84.0)	70 190 (16.0)
No	2 212 537 (91.1)	215 229 (8.9)
Emergency medical treatment due to injury during pregnancy		
Yes	53 307 (89.3)	6359 (10.7)
Once	51 033 (89.5)	6017 (10.5)
>Once	2274 (86.9)	342 (13.1)
No	2 527 905 (90.1)	279 060 (9.9)
Ever hospitalized due to injury during pregnancy		
Yes	3286 (84.3)	610 (15.7)
Once	3219 (84.4)	597 (15.6)
>Once	67 (83.75)	13 (16.25)
No	2 577 926 (90.1)	284 809 (9.9)

^a^ Includes anemia, heart disease, lung disease, diabetes, infections (eg, syphilis), gestational diabetes, excessive or insufficient amniotic fluid, high blood pressure, pregnancy toxemia, incomplete cervix, a history of producing newborns weighing at least 4000 g, a history of producing premature newborns or underweight infants, kidney disease, Rh factor allergy, autoimmune disease (eg, lupus erythematosus), prenatal bleeding, smoking during pregnancy, alcoholism, and drug addiction during pregnancy.

^b^ Includes fever, fecal material in amniotic fluid, early water breakage, early placental detachment, placenta previa, massive bleeding, spasms during childbirth, acute labor, prolonged labor, abnormal labor progression, breech or fetal position, cephalopelvic disproportion, complications related to umbilical cord prolapse, anesthesia, and fetal distress.

^c^ Includes amniocentesis, villi examination, labor induction, birth induction, fetal placement, cervical cerclage surgery, and laparotomy.

### Bivariate Analysis Results of Risk Factors for Adverse Pregnancy Outcomes

Table 3 presents the results of bivariate analysis of the association between risk factors and adverse pregnancy outcomes. Women receiving emergency treatments more than once (OR, 1.44; 95% CI, 1.31-1.59) had higher risks of adverse pregnancy outcomes compared with those receiving none. Women who experienced hospitalization more than once (OR, 1.78; 95% CI, 1.06-2.99) had higher risks of adverse pregnancy outcomes than those who did not experience hospitalization. Results for the covariates in this study were significant, such as age (20-34 years vs <20 years: OR, 1.38; 95% CI, 1.37-1.40; ≥35 years vs <20 years: OR, 1.41; 95% CI, 1.39-1.44), complications during labor (OR, 2.03; 95% CI, 2.01-2.04), and specific medical treatments during labor (OR, 1.70; 95% CI, 1.68-1.71).

**Table 3.** Bivariate Analysis Results Using Simple Logistic Regression

Independent variable	OR (95% CI)	
	Adverse pregnancy outcome	Premature delivery
Emergency visit due to injury		
Yes vs no	1.13 (1.11-1.16)	1.08 (1.05-1.11)
Once vs no	1.12 (1.09-1.14)	1.07 (1.04-1.10)
>Once vs no	1.44 (1.31-1.59)	1.36 (1.22-1.53)
Hospitalization due to injury		
Yes vs no	1.66 (1.54-1.80)	1.68 (1.54-1.83)
Once vs no	1.66 (1.54-1.80)	1.68 (1.54-1.83)
>Once vs no	1.78 (1.06-2.99)	1.77 (0.98-3.20)
Age, y		
≥35	1.41 (1.39-1.44)	1.34 (1.31-1.37)
20-34	1.38 (1.37-1.40)	1.51 (1.49-1.52)
<20	1 [Reference]	1 [Reference]
Income		
Low	1.15 (1.14-1.16)	1.14 (1.13-1.15)
Medium	1 [Reference]	1 [Reference]
High	0.94 (0.93-0.94)	0.92 (0.91-0.93)
No self-income	0.94 (0.93-0.95)	0.96 (0.95-0.97)
Urbanization level		
Rural, agricultural, or aging city	0.98 (0.97-0.98)	0.98 (0.97-0.99)
General	1.00 (0.99-1.01)	1.01 (1.002-1.02)
Emerging	1.03 (1.02-1.04)	1.02 (1.01-1.03)
Moderate	1.11 (1.09-1.12)	1.08 (1.07-1.10)
High	1 [Reference]	1 [Reference]
Nationality		
Taiwanese	0.72 (0.71-0.73)	0.70 (0.69-0.71)
Non-Taiwanese	1 [Reference]	1 [Reference]
Race		
Aboriginal	1.49 (1.46-1.51)	1.44 (1.41-1.47)
Non-Indigenous	1 [Reference]	1 [Reference]
Other diseases or substance use during pregnancy^a^		
Yes	1.50 (1.49-1.51)	1.61 (1.60-1.62)
No	1 [Reference]	1 [Reference]
Labor complications^b^		
Yes	2.03 (2.01-2.04)	2.23 (2.21-2.25)
No	1 [Reference]	1 [Reference]
Specific medical treatment requirements during labor^c^		
Yes	1.70 (1.68-1.71)	1.96 (1.94-1.98)
No	1 [Reference]	1 [Reference]

Abbreviation: OR, odds ratio.

^a^ Includes anemia, heart disease, lung disease, diabetes, infections (eg, syphilis), gestational diabetes, excessive or insufficient amniotic fluid, high blood pressure, pregnancy toxemia, incomplete cervix, a history of producing newborns weighing at least 4000 g, a history of producing premature newborns or underweight infants, kidney disease, Rh factor allergy, autoimmune disease (eg, lupus erythematosus), prenatal bleeding, smoking during pregnancy, alcoholism, and drug addiction during pregnancy.

^b^ Includes fever, fecal material in amniotic fluid, early water breakage, early placental detachment, placenta previa, massive bleeding, spasms during childbirth, acute labor, prolonged labor, abnormal labor progression, breech or fetal position, cephalopelvic disproportion, complications related to umbilical cord prolapse, anesthesia, and fetal distress.

^c^ Includes amniocentesis, villi examination, labor induction, birth induction, fetal placement, cervical cerclage surgery, and laparotomy.

### Bivariate Analysis Results of Risk Factors for Premature Delivery

Table 3 lists the results of bivariate analysis of risk factors associated with premature delivery. We obtained results similar to those for adverse pregnancy outcomes. Women who required emergency treatment related to injuries had an 8% increased probability of premature delivery (OR, 1.08; 95% CI, 1.05-1.11) compared with those who did not. A similar result was obtained for women who experienced injury-related hospitalization (OR, 1.68; 95% CI, 1.54-1.83). We also found similar results for our covariates, for age (20-34 years vs <20 years: OR, 1.51; 95% CI, 1.49-1.52; ≥35 years vs <20 years: OR, 1.34; 95% CI, 1.31-1.37), income (low vs medium income: OR, 1.14; 95% CI, 1.13-1.15), with labor complications (OR, 2.23; 95% CI, 2.21-2.25), and with other diseases or substance use during pregnancy (OR, 1.61; 95% CI, 1.60-1.62). No multicollinearity was found.

### Multivariate Analysis Results of Risk Factors for Adverse Pregnancy Outcomes

Table 4 shows the results of the multiple logistic regression model for adverse pregnancy outcomes. In model 1, women who received emergency treatment for injuries were more likely to have adverse pregnancy outcomes (AOR, 1.08; 95% CI, 1.05-1.10) after controlling for other risk factors. Model 2 showed that women who received more than 1 emergency treatment because of injuries had a higher risk of adverse pregnancy outcomes (AOR, 1.35; 95% CI, 1.22-1.49) than did those who required none. In model 3, after other risk factors were controlled for, women with injury-related hospitalization had a 53% higher probability of adverse pregnancy outcomes (AOR, 1.53; 95% CI, 1.41-1.65) than those without it.

**Table 4.** Multivariable Analysis Result Using Multiple Logistic Regression Adjusted by All Covariates

Independent variable^a^	AOR (95% CI)	
	Adverse pregnancy outcome	Premature delivery
Emergency visit due to injury		
Yes vs no	1.08 (1.05-1.10)	1.03 (0.96-1.12)
Once vs no	1.07 (1.04-1.09)	1.02 (0.94-1.10)
>Once vs no	1.35 (1.22-1.49)	1.44 (1.05-1.97)
Hospitalization due to injury		
Yes vs no	1.53 (1.41-1.65)	2.33 (1.90-2.87)
Once vs no	1.53 (1.41-1.65)	2.23 (1.80-2.76)
>Once vs no	1.61 (0.95-2.74)	6.72 (2.86-15.80)

Abbreviation: AOR, adjusted odds ratio.

^a^ Adjusted for age, income, urbanization level, nationality, race, other diseases or substance use during pregnancy, labor complications, and specific medical treatment during pregnancy.

### Multivariable Analysis Results of Risk Factors for Premature Delivery

Table 4 provides the results from the multiple logistic regression model for premature delivery. According to model 1, no association was found between the requirement for emergency treatment due to injury and premature delivery (AOR, 1.03; 95% CI, 0.96-1.12) after controlling for other risk factors. In model 2, women who received more than 1 emergency treatment due to injury had a 1.44 times higher risk of premature delivery (AOR, 1.44; 95% CI, 1.05-1.97) than those who received none. In model 3, women who experienced injury-related hospitalization had a 133% higher risk of premature delivery (AOR, 2.33; 95% CI, 1.90-2.87) than those who experienced none. A significant association also existed between hospitalization once (AOR, 2.23; 95% CI, 1.80-2.76) or 2 or more times (AOR, 6.72; 95% CI, 2.86-15.80) and premature delivery.

## Discussion

One of the key results in this study is that women who had emergency department visits or hospitalization due to injuries were more likely to have adverse pregnancy outcomes, including premature delivery, after controlling for demographic and clinical risk factors. This finding corroborates those of other studies indicating that women have higher risks of adverse pregnancy outcomes, such as premature delivery and preterm birth, if they are hospitalized or treated at an emergency department because of injury.^9,10,19^ Such effects may be caused by increased risks of placental abruption, uterine rupture, and fetal distress due to injury. Studies have suggested that women who experience injury-related hospitalization have higher risks of placental abruption, uterine rupture, and fetal distress.^9,10^ Research has also concluded that women who receive treatment at emergency departments due to injury have higher risks of placental abruption and uterine rupture.^19^ These maternal conditions could increase risks of adverse outcomes, such as premature delivery, birth defects, low birth weight, and stillbirth.^21,22^ Therefore, pregnant women sustaining minor or major injuries should be treated carefully by a multidisciplinary team that incorporates a trauma surgeon and an obstetrician.

In accordance with other studies, this study found that women who receive specific medical treatments, such as birth induction or labor induction, are more likely to have adverse pregnancy outcomes, including premature delivery.^23,24,25^ This finding can be explained by the increased risk of adverse effects, such as hemorrhage and infection, from the induced termination of pregnancy. Relevant literature has shown that the induced termination of pregnancy is also associated with an increased risk of hemorrhage and infection among pregnant women.^26^

Our finding is consistent with those of studies indicating that women with labor complications, such as placenta previa, fetal distress, or massive bleeding, have increased probabilities of adverse pregnancy outcomes, including premature delivery.^21^ Our result could be attributed to the increased risk of placental abruption during pregnancy owing to labor complications. Results of the study by Tikkanen et al^24^ support our results, indicating that placenta previa and massive bleeding were apparent risk factors for placental abruption. Early detection of labor complications at prenatal care visits could reduce the risk of adverse pregnancy outcomes.

Our finding is supported by research indicating that women with other diseases or engagement in substance use, such as history of premature birth, prenatal bleeding, smoking, gestational diabetes, infectious diseases, and alcohol use, are more likely to have adverse pregnancy outcomes, including premature delivery.^27,28,29^ This result may be because of increased risks of placental abruption and placenta previa among women who smoke, drink alcohol, and experience prenatal bleeding.^21,22,30^ Both placental abruption and placenta previa are common risk factors for adverse pregnancy outcomes.^21,22^

In this study, advanced and young maternal age were associated with an increased risk of adverse pregnancy outcomes, including premature delivery, which is consistent with the results of other studies.^31,32,33,34,35,36,37^ This finding may be because of prepregnancy obesity and an increased rate of fetal distress and preeclampsia associated with advanced maternal age, as suggested by other studies.^33,34,35^ Other studies have supported our finding that an increased rate of complications during pregnancy may influence the risk of adverse pregnancy outcomes among young women.^36,37^

Finally, our result is consistent with those of studies that have found that women with low incomes exhibit higher risks of adverse pregnancy outcomes^38,39,40,41,42,43^; they may have access only to poor prenatal care and may have little access to health facilities. Research using data from the Indonesia Demographic and Health Survey concluded that the low household wealth index was associated with the underuse of prenatal care.^44^ Furthermore, women with poor prenatal care had higher risks of adverse pregnancy outcomes.^45^

Our main finding pertains to the association between injury and adverse pregnancy outcomes. Both hospitalization and emergency visits owing to injury were associated with adverse pregnancy outcomes. The strengths of this study include the use of a nationwide data set with multiple sources of data constituting prospectively collected data and comprehensive clinical data.

### Limitations

This study has several limitations. It was restricted to the variables for which data were available, including environment; prenatal care visit (including frequency and treatment given during visit), education level, and paternity; prehospital information; and injury mechanism. Although the 3 data sets we used encompass a wide range of variables, other variables, such as motor vehicle collision configuration, prehospital care, and prenatal care, which could play a crucial role in determining adverse pregnancy outcomes, are not readily available. This study used national-level data in Taiwan. The results obtained can be generalized to countries with conditions similar to Taiwan’s. Nonetheless, caution should be exercised in generalizing them to other settings.

## Conclusions

This study found that pregnant women who experience injury had higher risks of adverse pregnancy outcomes, including premature delivery. Both hospitalization and emergency department visits due to injury were associated with an increased risk of adverse pregnancy outcomes. Therefore, pregnant women sustaining minor or major injuries should be treated carefully by a multidisciplinary team including a trauma surgeon and an obstetrician.
